# Supplementary material for: A Plasmid Carrying blaIMP-56 in Pseudomonas aeruginosa Belonging to a Novel Resistance Plasmid Family
Source: Microorganisms. 2022 Sep 17;10(9):1863. doi: 10.3390/microorganisms10091863 (PMC9501424; doi:10.3390/microorganisms10091863)
Supplement: Supplementary file 1 [file microorganisms-10-01863-s001.zip › Supplementary material.pdf]

Table S1. Characteristics of the plasmids included in the phylogenetic tree of the RepA proteins.

Plasmid name	Strain	Accession number	Country	Size (bp)	Resistance genes	Replicase	Family	Incompatibility group
unnamed1	AR_0353	NZ_CP027173.1	-	41,559	bla_{GES-1}	RepA	*PriCT-1 superfamily*	ND
unnamed1	AR_0356	NZ_CP027168.1	-	57,053	bla_{KPC-2}	RepA	*Rep_3 superfamily*	ND
unnamed2	AR_0356	NZ_CP027170.1	-	438,531	*aadA16, tet(G), qacE*Δ1, *aac(6')-Ib4*	RepA	*Rep_3 superfamily*	ND
unnamed3	AR441	NZ_CP029094.1	-	438,529	*aac(6')-Ib4, aadA16, tet(G), sul1, qacE*Δ1	RepA	*Rep_3 superfamily*	ND
p1	C79	NZ_CP040685.1	China	40,180	bla_{KPC-2}	RepA	*HTH superfamily*	IncP-6
pF092021-2	F092021	NZ_CP081289.1	China: Henan	32,558	*aac(6')-ll*, bla_{IMP-10}	RepA	*Rep_3 superfamily*	ND
unnamed1	P8W	CP081478.1	China: Tianjin	232,884	*sul1, cmlA6, qacE, ant(2")-la*	RepA	*Rep_3 superfamily*	ND
pOZ176	PA96	NC_022344.1	Guangzhou, China	500,839	*aac(6')-lb4, aac(6')-lb4, aac(6')-lb4, aph(3')-II, sul1, catB8a*, bla_{OXA-10}, bla_{IMP-9}, *qacE*Δ1	RepA	*Rep_3 superfamily*	IncP-2
pMATVIM-7	P07-406	AM778842.1	USA: Texas	24,179	bla_{VIM-7}	RepA	*HTH superfamily*	ND
pNOR-2000	COL-1	NC_020452.1	France	21,880	*qacE*Δ1, *sul1*, bla_{VIM-2}	RepA	*Rep_3 superfamily*	ND

pCOL-1	ST308	NC_022346.1	Colombia	31,529	*bla_{KPC-2}*	RepA	*HTH superfamily*	IncP-6
unnamed	FDAARGOS_570	CP033834.1	USA	36,032	*aac(6′)-lb3, aph(6)-ld, aph(3′′)-Ib, aac(6′)-lb3, sul1, bla_{OXA-10}, bla_{VIM-6}, bla_{OXA-10}, qacEΔ1, cmx*	RepA	*HTH superfamily*	ND
p10265-KPC	10265	NZ_KU578314.1	China	38,939	*bla_{TEM}, bla_{KPC-2}*	RepA	*HTH superfamily*	IncP-6
pCB58	-	NZ_KY630469.1	Porto, Portugal	32,207	*aac(6′)-lb4, aac(3)-Ia, aac(6′)-Il, aph(6)-ld, aph(3′′)-Ib, qacEΔ1, sul1, bla_{VIM-2}*	RepA	*Rep_3 superfamily*	ND
pAMBL2	PAO1	NZ_KP873171.1	Madrid	24,133	*aadA1, aac(6′)-lb4, sul1, bla_{VIM-1}, bla_{VIM-1}, bla_{VIM-1}, qacEΔ1*	RepA	*Rep_3 superfamily*	ND
pJB37	FFU NA P_PS_37	NZ_KY494864.1	Porto, Portugal	464,804	*aac(6′)-Il, aac(3)-Ia, aac(6′)-Ib4, qacEΔ1, sul1, bla_{VIM-2}*	RepA	*Rep_3 superfamily*	IncP-2
pD5170990	D5170990	KX169264.1	Brazil	32,424	*sul1, aac(6′)-ll, aph(3″)-lb, aph(6)-ld, qacEΔ1, cmx, bla_{KPC-2}*	RepA	*HTH superfamily*	ND

p243931-IMP	243931	NZ_MN208062.1	China; Zhejiang	392,046	*msr(E), armA, aph(3′)-Ia, aac(6′)-lb4, ARR-2, mph(E), qnrVC1, sul1, dfrA22, tet(C), bla*$_{OXA-1}$, *bla*$_{IMP-45}$, *qacE*Δ1, *catB3*	RepAcin	COG5527 superfamily	ND
p60503-DIM	60503	NZ_MN208063.1	China: Beijing	407,628	*catB3, qacE*Δ1, *aac(6′)-IIa, aac(6′)-IIa, aac(6′)-lb, aph(3″)-lb, aadA1, aph(6)-ld, qnrVC6, sul1, dfrA1, dfrB, bla*$_{OXA-4}$, *bla*$_{DIM}$	RepA	*AAA_25 ATPase*	ND
pJB35	-	NZ_MF168945.1	Portugal	31,166	*aac(6′)-lb4, aac(6′)-ll, qacE*Δ1, *sul1, bla*$_{VIM-2}$	RepA	*Rep_3 superfamily*	ND
pCOOP-101	PAcoop101	NZ_MF141039.1	United kingdom	26,108	*dfrA1, sul1, aadA22, aac(6′)-ll, qacE*Δ1, *bla*$_{VIM-4}$	RepA	*Rep_3 superfamily*	ND
pOXA-198	PA41437	NZ_MG958650.1	France	48,978	*cmlA1, qacE*Δ1, *sul1, bla*$_{OXA-198}$, *aac(6′)-ll*	RepA	*PriCT-1 superfamily*	ND
pPA2047	2047	NZ_MN082782.1	Argentina	43,660	*bla*$_{KPC-2}$	RepA	*PriCT-1 superfamily*	ND

pTROUS1	163940	NZ_MK047610.1	France	42,035	*aac(6′)-lb, qacEΔ1, sul1, bla*$_{OXA-19}$, *bla*$_{IMP-63}$	RepA	*Rep_3 superfamily*	ND
pMRVIM0713	MRSN17623	KP975076.1	USA	36,032	*aac(6′)-lb3, aac(6′)-lb3, aph(6)-ld, aph(3′′)-Ib, sul1, bla*$_{OXA-10}$, *bla*$_{VIM-6}$, *qacEΔ1, cmx*	RepA	*HTH superfamily*	ND
pNK546b	NK546	NZ_MN583270.1	China: Tianjin	232,884	*sul1, qacE, cmlA6, ant(2″)-la*	RepA	*Rep_3 superfamily*	IncP-7
p1160-VIM	1160	NZ_MF144194.1	China	205,426	*msr(E), aph(3″)-lb, aph(6)-Id, aac(6′)-lb4, aac(6′)-Ib, dfrA42, aadA1, mph(E), ere(A), qnrVC6, sul1, bla*$_{VIM-24}$, *qacEΔ1, catB3*	RepA	*Rep_3 superfamily*	IncP-7
p2	ZPPH2	NZ_CP077987.1	China: Hangzhou	35,358	*tet(A)*	RepA	*Rep_3 superfamily*	ND
p4	ZPPH1	NZ_CP077992.1	China: Hangzhou	35,358	*tet(A)*	RepA	*Rep_3 superfamily*	ND
pFAHZU40-KPC	FAHZU40	NZ_CP078008.1	China: Hangzhou	28,700	*aac(6′)-IbΔ, aac(6′)-IbΔ, qnrVC4, cmlA5,bla*$_{TEM-1C}$, *bla*$_{KPC-2}$	RepA	*Rep_3 superfamily*	ND
pQZPH41-KPC	QZPH41	NZ_CP064400.1	China: Quzhou	88,210	*bla*$_{KPC-2}$	RepA	*Rep_3 superfamily*	ND

pPUV-1	NMI804/03	NZ_MT732179.1	Poland	489,508	*aadA10, aadA10, aadA10, aadA10, qacE*Δ1, *sul1, bla*$_{VIM-2}$, *bla*$_{VIM-2}$, *bla*$_{VIM-2}$, *bla*$_{OXA-2}$, *ant(2”)-la, ant(2”)-la, ant(2”)-la*	RepA	*Rep_3 superfamily*	IncP-2

To determine the country of isolation we relied on the information of the submitters in GenBank. Δ symbol indicates a truncated gen.

Table S2. Characteristics of *P. aeruginosa* plasmids with similar structure like pPE52IMP.

Strain	Plasmid name	Accession Number	Country	Source	Size (bp)	Resistance genes
FDAARGOS_570	unnamed	CP033834.1	USA	Collection	36,032	*aph(3′′)-Ib*, *aph(6)-Id*, *aac(6′)-Ib3*, *aac(6′)-Ib3*, *sul1*, bla_{VIM-6}, bla_{OXA-10}, bla_{OXA-10}, *qacE*Δ1, *cmx*
D5170990	pD5170990	KX169264.1	Brazil	Blood	32,424	*cmx*, *qacE*Δ1, *sul1*, bla_{KPC-2}, *aph(6)-Id*, *aac(6′)-Il*, *aph(3′′)-Ib*
MRSN17623	pMRVIM0713	KP975076.1	USA	Unknown	36,032	*aph(3′′)-Ib*, *aac(6′)-Ib3*, *aac(6′)-Ib3*, *aph(6)-Id*, *sul1*, bla_{VIM-6}, bla_{OXA-10}, bla_{OXA-10}, *qacE*Δ1, *cmx*
P07-406	pMATVIM-7	AM778842.1	USA: Texas	Clinical isolate	24,179	bla_{VIM-7}
164130	p4130-KPC	MN336501.1	France	Urine	58,104	*aac(6′)-Ib4*, *sul1*, bla_{KPC-2}, bla_{KPC-2}, *qacE*Δ1, *catB3*, $bla_{OXA-732}$, $bla_{OXA-779}$

Table S3. Characteristics of plasmids from other bacterial genera with similar structure like pPE52IMP.

Strain	Plasmid name	Accession Number	Country	Source	Size (bp)	Resistance genes
138R	p138R	MK423762.1	Argentina	Patient with cystic fibrosis	34,096	*aac(6′)-Ib4*, *qacE*Δ1, *sul1*
SM39	pSMC1	AP013064.1	Japan	Clinical isolate	41,517	*aadA2*, *qacE*Δ1, *sul1*, *bla*$_{IMP-1}$, *bla*$_{CMY-8}$

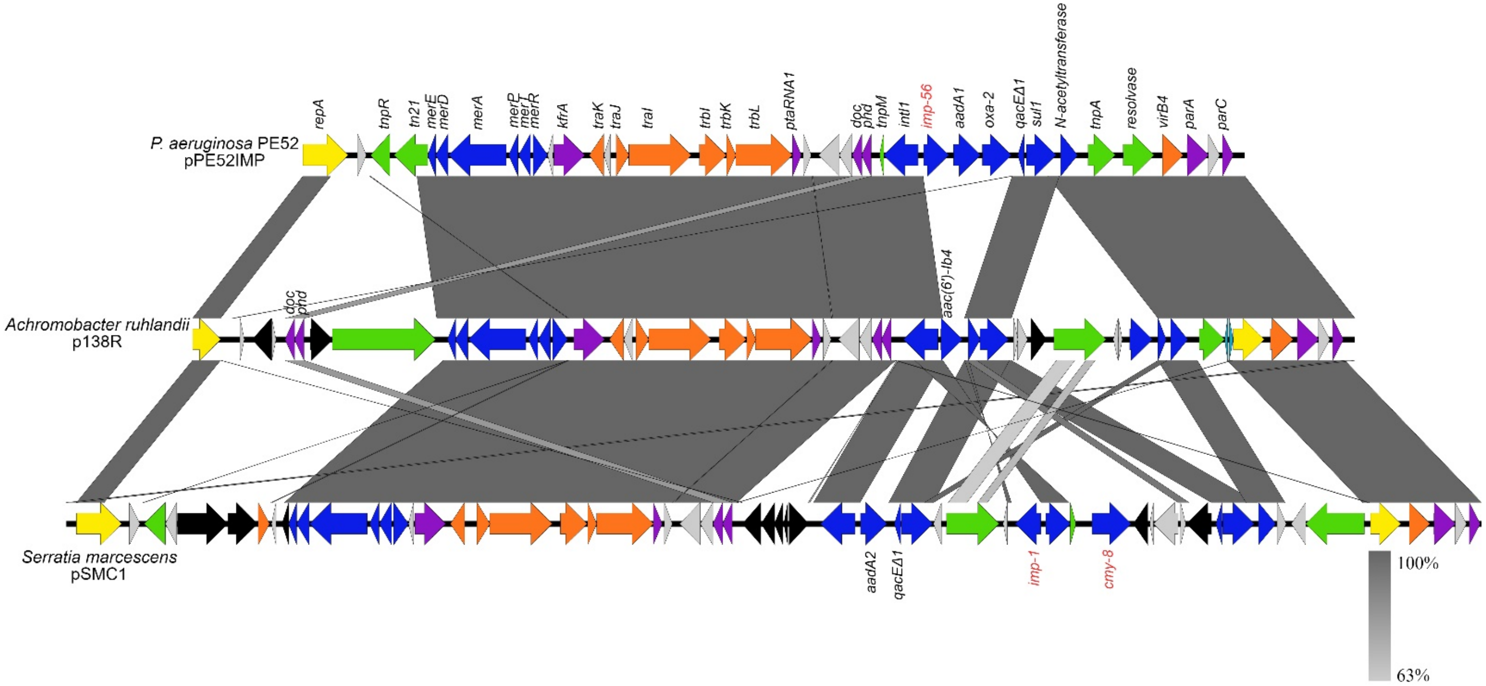

Figure S1. Comparison of pPE52IMP with plasmids from other bacterial genera with similar backbone. This figure was built with Easyfig [44]. Plasmid modules were represented with different colors. Yellow: replication; Purple: stability; Orange: mobilization; Blue: adaptation; Green: transposons; Grey: hypothetical proteins; Black: other genes. The gray color level indicates the percentage of BLAST identity of the plasmids. p138R (accession no. MK423762.1), pSMC1 (accession no. AP013064.1).
