# Supplementary figures and images for: A Plasmid Carrying blaIMP-56 in Pseudomonas aeruginosa Belonging to a Novel Resistance Plasmid Family
Source: Microorganisms. 2022 Sep 17;10(9):1863. doi: 10.3390/microorganisms10091863 (PMC9501424; doi:10.3390/microorganisms10091863)

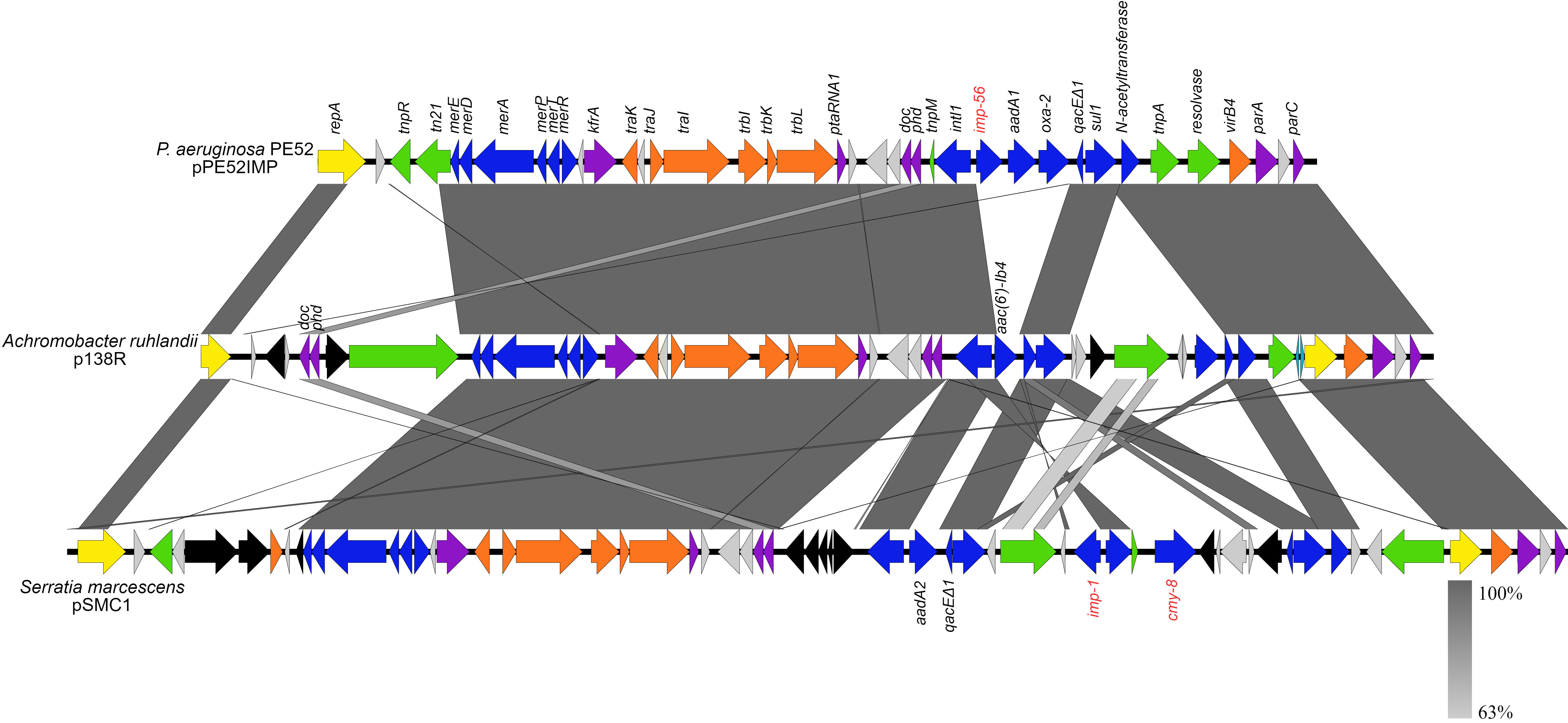

Supplement: Supplementary file 1 [file microorganisms-10-01863-s001.zip › Figure S1.jpeg]
